# Mechanism Exploration of the Effect of Polyamines on the Polishing Rate of Silicon Chemical Mechanical Polishing: A Study Combining Simulations and Experiments

**DOI:** 10.3390/nano14010127

**Published:** 2024-01-04

**Authors:** Ziwei Lin, Junli Zhu, Qi Huang, Lei Zhu, Weimin Li, Wenjie Yu

**Affiliations:** 1 National Key Laboratory of Materials for Integrated Circuits, Shanghai Institute of Microsystem and Information Technology, Chinese Academy of Sciences, Shanghai 200050, China; linzw@mail.sim.ac.cn (Z.L.); huangqi@mail.sim.ac.cn (Q.H.); weimin.li@mail.sim.ac.cn (W.L.); casan@mail.sim.ac.cn (W.Y.); 2 College of Materials Science and Opto-Electronic Technology, University of Chinese Academy of Sciences, Beijing 100049, China; 3 Shanghai Institute of IC Materials Co., Ltd., Shanghai 201899, China; junli.zhu@sicm.com.cn

**Keywords:** chemical mechanical polishing, silicon wafer, polyamines, adsorption mechanism, material removal rate, ReaxFF molecular dynamics simulation

## Abstract

Polyamines have become important chemical components used in several integrated circuit manufacturing processes, such as etching, chemical mechanical polishing (CMP), and cleaning. Recently, researchers pointed out that polyamines can be excellent enhancers in promoting the material removal rate (MRR) of Si CMP, but the interaction mechanism between the polyamines and the silicon surface has not been clarified. Here, the micro-interaction mechanisms of polyamines, including ethylenediamine (EDA), diethylenetriamine (DETA), triethylenetetramine (TETA), tetraethylenepentamine (TEPA), and pentaethylenehexamine (PEHA), with the Si(1, 0, 0) surface were investigated through molecular dynamics (MD) simulations using the ReaxFF reactive force field. Polyamines can adsorb onto the Si(1, 0, 0) surface, and the adsorption rate first accelerates and then tends to stabilize with the increase in the quantity of -CH_2_CH_2_NH-. The close connection between the adsorption properties of polyamines and the polishing rate has been confirmed by CMP experiments on silicon wafers. A comprehensive bond analysis indicates that the adsorption of polyamines can stretch surface Si–Si bonds, which facilitates subsequent material removal by abrasive mechanical wear. This work reveals the adsorption mechanism of polyamines onto the silicon substrate and the understanding of the MRR enhancement in silicon CMP, which provides guidance for the design of CMP slurry.

## 1. Introduction

Silicon (Si) wafers are the predominant base substrate materials for the manufacture of semiconductor devices in the field of integrated circuits (ICs) [1,2,3], and their processing quality directly affects device performance. Chemical mechanical polishing (CMP) is the key processing part of the Si wafers’ manufacturing, enabling global flattening [4,5,6,7]. Recently, new device structures and advanced process technologies have been developed to achieve rapid improvements in the integration and performance of ICs, resulting in an urgent need to enhance the surface quality of Si wafers. Therefore, CMP technology for Si wafer manufacturing is required to achieve both higher material removal rate (MRR) [8,9] and lower surface roughness.

**Figure 1:**
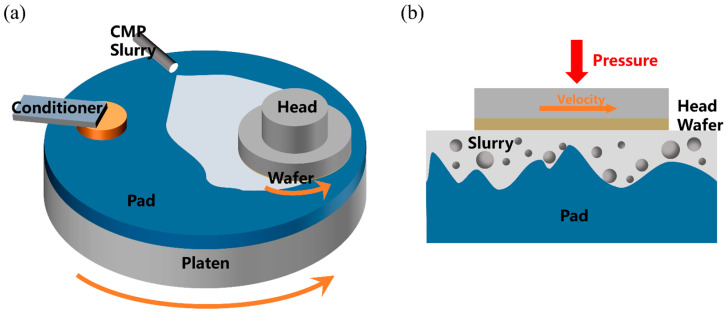
(**a**) Diagram of the CMP process for a silicon wafer, and (**b**) the three-body (polishing pad, abrasives, and silicon wafer) wear mechanism in the CMP process.

Si CMP typically utilizes the synergistic effect of chemical reactions and mechanical wear to achieve material removal [10,11,12,13,14]. During this process, the polishing slurry is transported to the area between the wafer and the pad, where the OH^−^ reacts chemically with the Si wafer, forming a softened layer, and then this layer is quickly removed by the three-body mechanical friction among the abrasive particles, the polishing pad, and the slurry (as shown in Figure 1). Thus, polishing slurry is an essential component of CMP technology and a key factor in improving the removal rate. Recently, to achieve a higher MRR, some specific additives have been selected and added to the polishing slurry. Song et al. have reported that the strong oxidant H_2_O_2_ can improve the MRR of Si CMP, with the most significant effect at a concentration of 1 vol% [15]. Xie et al. pointed out that cations such as Na^+^, K^+^, NH_4_^+^, etc., are able to increase the MRR of Si CMP [16,17] while allowing the slurry to maintain good dispersion. Additionally, sorbitol, gluconic acid, citric acid, and ammonium citrate were found to be effective in improving the removal rate of Si CMP, particularly ammonium citrate [18]. Experiments conducted by Xie et al. showed that the slurry containing 20 mmol of ammonium citrate caused a 71.6% increase in the MRR compared to the control group.

Compared to the above additives, new additives with amine groups have also become popular additives for silicon wafer CMP, due to the following advantages: (1) they can achieve a higher MRR (>500 nm/min) [19,20], (2) they do not introduce contamination with metal ions such as Na^+^, K^+^, etc., and (3) they can be used as chelating agents to remove metal ions [21,22]. Polyamines are a representative class of amine additives that can achieve large MRR improvements. Beo et al. [23] achieved an MRR of up to 552.8 nm/min using polyamines in the Si CMP process, which is three times higher than the MRR of conventional metallic alkalis such as KOH and NaOH. Researchers have investigated the role of polyamines in Si CMP using X-ray photoelectron spectroscopy (XPS) and contact angle testing [23,24]. They discovered that polyamine chemically reacts with the silicon surface to form Si–N bonds, and that it can effectively reduce the contact angle of slurry on the silicon surface. However, due to experimental limitations, it is not possible to observe the chemical reaction process between polyamine and the silicon wafer surface at a microscopic level, leaving the mechanism of polyamine in Si CMP not entirely clear. Exploring and understanding the microscopic mechanism of interaction between polyamines and silicon wafers is beneficial for the design of novel additives used in Si CMP.

Computational simulation methods, such as quantum chemical calculation based on density functional theory (DFT) and MD simulation, have been widely used to study interfacial interactions in the field of chemical mechanical polishing [25,26,27,28,29,30]. However, quantum chemical calculation is limited by computing power and cannot accurately simulate large systems. Traditional MD simulations based on classical force fields can simulate the dynamic evolution of large systems with thousands or even tens of thousands of atoms, but they have the disadvantage of not being able to describe the chemical reactions. The ReaxFF reactive force field [31] can compensate for this inability of the classical force field by describing the reactions between atoms in terms of bond distance and bond order. Based on this advantage, ReaxFF MD simulations have already been successfully applied to study the material removal mechanism during the Si CMP process [32,33,34,35,36,37]. Therefore, the current ReaxFF MD method is suitable for the research of the micro-mechanisms of polyamines in this study.

Here, we explored the micro-interaction mechanism of the polyamines with the Si(1, 0, 0) surface through an MD simulation method based on the ReaxFF reactive force field. We studied the adsorption behavior of the polyamines, including ethylenediamine (EDA), diethylenetriamine (DETA), triethylenetetramine (TETA), tetraethylenepentamine (TEPA), and pentaethylenehexamine (PEHA), and then carried out the corresponding CMP experiments in order to correlate their adsorption features with their effectiveness in enhancing the MRR. Finally, a mechanistic explanation was provided for the MRR improvement with these polyamines, which can provide a theoretical guide for CMP slurry design.

## 2. Materials and Methods

### 2.1. Model Construction

**Figure 2:**
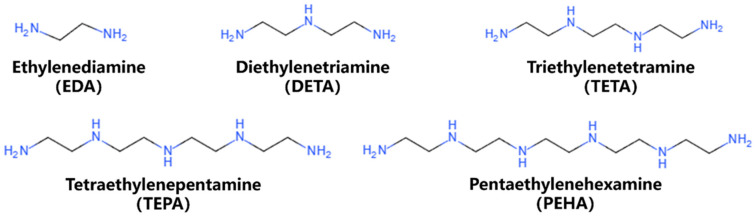
Molecular structures of the five polyamines.

In order to simulate the interaction between the polyamine molecules and the silicon wafer, a layer-by-layer simulation model was constructed containing two parts: the Si(1, 0, 0) substrate and the molecular layer. The main procedure for building the model was as follows: (1) A Si layer about 15 Å thick was cut from the bulk silicon, the top surface of which was the (1, 0, 0) crystal plane. Then, the Si layer was expanded along the x- and y-directions into a 54.3 × 54.3 Å^2^ slab, composed of 2400 Si atoms. The Si substrate was relaxed under the NPT ensemble for 1 ns to bring the structure close to that at 1 atm pressure. The final size of the Si substrate was 53.283 × 53.282 × 14.937 Å^3^. The lower region of about 7 atomic layers (~9 Å) of the Si substrate was fixed as the ‘Rigid Layer’, while the upper region of about 5 atomic layers (~6 Å) was defined as the ‘Movable Layer’. This model setup can meet the requirements of adsorption simulations and effectively reduces the computational workload. Subsequently, a relaxation process was conducted under the NVT ensemble to bring the ‘Movable Layer’ to an equilibrium state. (2) The molecular structure of each type of polyamine was constructed using BIOVIA Materials Studio, and the geometry optimization was carried out through the DFT method. For unimolecular adsorption simulations, a layer containing only a centrally located single polyamine molecule was created. For multimolecular simulations, the molecular layer with a certain number of molecules was generated using Packmol [38] software (version GENCAN). The molecular structures of the five polyamines studied in this work are shown in Figure 2.

**Figure 3:**
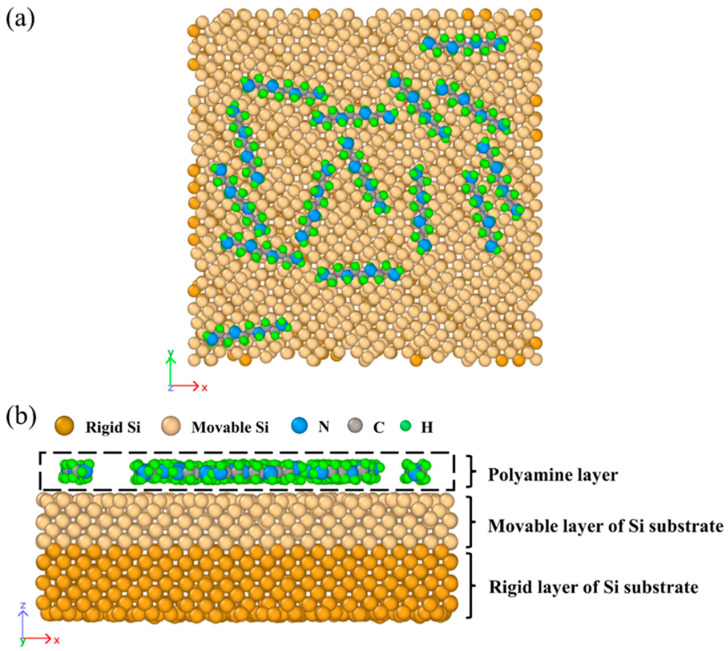
The interaction model containing the polyamine layer and the Si substrate: (**a**) top view and (**b**) side view.

Once the above procedure was completed, the prepared Si(1, 0, 0) substrate and the molecular layer were combined together to form a stacked structure, maintaining a 3.5 Å spacing. Such spacing allows for attractive interactions between the two stacked layers, preventing the molecular layer from behaving like gas diffusion. Figure 3 shows an example model for simulating the multimolecular adsorption of 15 TETA molecules on a silicon surface. There are a total of three layers in the detailed model: the rigid Si layer, the movable Si layer, and the molecular layer. The constructed model was subjected to an MD simulation with the NVT ensemble to investigate the microscopic interactions between polyamines and the silicon wafer. Throughout the entire simulation, the rigid Si atoms on the bottom remained fixed, while the atoms from the Si surface and the molecular layer were allowed to move.

### 2.2. Simulation Details

All of the MD simulations for the present work were performed using the Large-scale Atomic/Molecular Massively Parallel Simulator (LAMMPS) [39,40] with the Si/C/N/H/O [41] ReaxFF force field. This ReaxFF force field was recently developed by Bhati et al. as a combination of the existing C/N/H and Si/C/H force field parameters with the addition of the Si/N/H force field parameters. Bhati has used this force field to research the adsorption characteristics of polymer binders on the Si anode surface in lithium-ion batteries. In addition, the simulations were run under NVT or NPT ensembles with the default Nosé–Hoover method for both the thermostat and the barostat. The time step was set to 0.25 fs. The temperature of the system was controlled at 300 K, and the Verlet algorithm was used as the integrator. The adsorption motion of polyamines occurs in the z-direction, so the boundary conditions in the z-direction were set to be non-periodic, while those in the x- and y-directions were set to be periodic. The simulation time for unimolecular adsorption was 500 ps to ensure complete adsorption. Meanwhile, due to the increased computational cost of the multimolecular adsorption simulation, the simulation time was reduced to 100 ps on the premise of ensuring effective statistics of adsorption time. Finally, the atomic trajectories obtained from the MD simulations were processed by means of VMD-1.9.3 [42] and OVITO-3.7.12 [43] software.

### 2.3. Experimental Details

**Figure 4:**
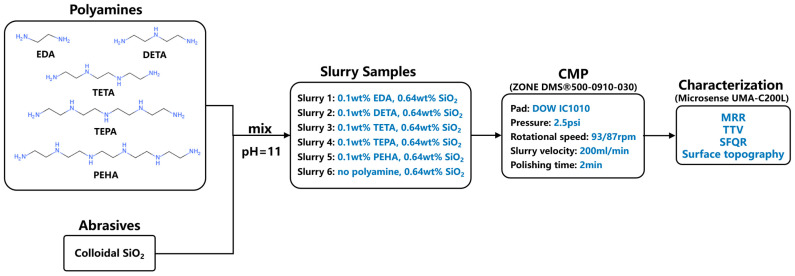
The schematic diagram of the experimental procedure.

The CMP experiments were carried out on commercially available N-type (1, 0, 0) silicon wafers. Five types of CMP slurry samples were prepared by mixing pure colloidal SiO_2_ abrasive (FUSO CHEMICAL CO., LTD, Tokyo, Japan) with EDA (Aladdin, Shanghai, China, 98%), DETA (Aladdin, Shanghai, China, 99%), TETA (Aladdin, Shanghai, China, 70%), TEPA (Aladdin, Shanghai, China, technical grade), and PEHA (Sigma-Aldrich, Amsterdam, Netherlands, technical grade), respectively. The concentrations of the polyamines in the CMP slurries were fixed at 0.1 wt%, and the pH of the slurry was adjusted to 11.0 using a KOH solution (Aladdin, Shanghai, China, 50% solution). The reference slurry only contained the same concentration of colloidal SiO_2_ abrasive, without the addition of polyamine. KOH solution was also used to adjust the pH of the reference slurry to 11.0. As shown in Figure 4, the above slurry samples were named Slurry 1–6, respectively.

All of the CMP experiments were performed on an 8-inch wafer CMP polisher (SIZONE DMS^®^500-0910-030, SIZONE Tech. Inc., Hangzhou, China) with a polyurethane polishing pad (DOW IC1010, DOW Inc., Hsinchu, Taiwan, China), and four dummy wafers were polished for equipment debugging prior to the actual testing of the CMP slurry. The process parameters used for the CMP experiments are listed in Figure 4. During the polishing process, the pressure applied to the polishing head was 2.5 psi, and the rotation speeds of the polishing head and the plate were set to 93 and 87 rpm, respectively. The flow rate of the CMP slurry was 200 mL/min. The polishing time for each wafer was 2 min, and all wafers were rinsed under DI water and dried after CMP. The wafers’ thicknesses before and after polishing were determined using a measuring machine (Microsense UMA-C200L, KLA Co., Tempe, AZ, USA) to calculate the material removal rate during the polishing process, and the flatness of the wafers was also characterized by this equipment. The thickness measurement accuracy of the Microsense UMA-C200L is 0.001 µm.

## 3. Results and Discussion

### 3.1. Unimolecular Adsorption of Polyamine

**Figure 5:**
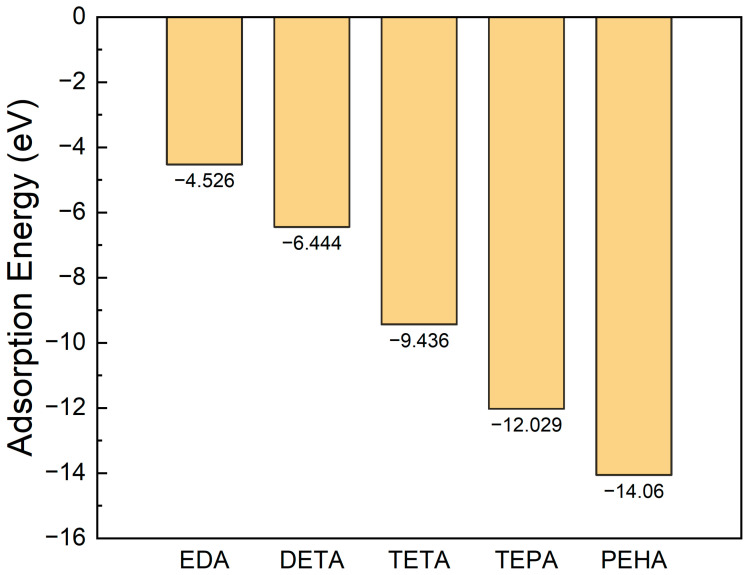
The adsorption energies of the five types of polyamines (EDA, DETA, TETA, TEPA, and PEHA) on the Si(1, 0, 0) surface.

To understand the adsorption behavior of polyamines on the silicon surface, unimolecular adsorption simulations of each polyamine were carried out. Figure 5 shows the adsorption energy of each polyamine on the Si surface. It can be seen that the adsorption energy of the polyamine increases almost linearly from 4.526 to 14.060 eV as the chain length and the quantity of -CH_2_CH_2_NH- increases. From the bonding information output from the MD simulations, it can be seen that all of the N atoms in each molecule are bonded with the surface Si atoms, as judged by the Si/C/N/H/O ReaxFF force field, implying that amine group is the main adsorption site and that its adsorption on the Si surface contributes to the increase in adsorption energy.

**Figure 6:**
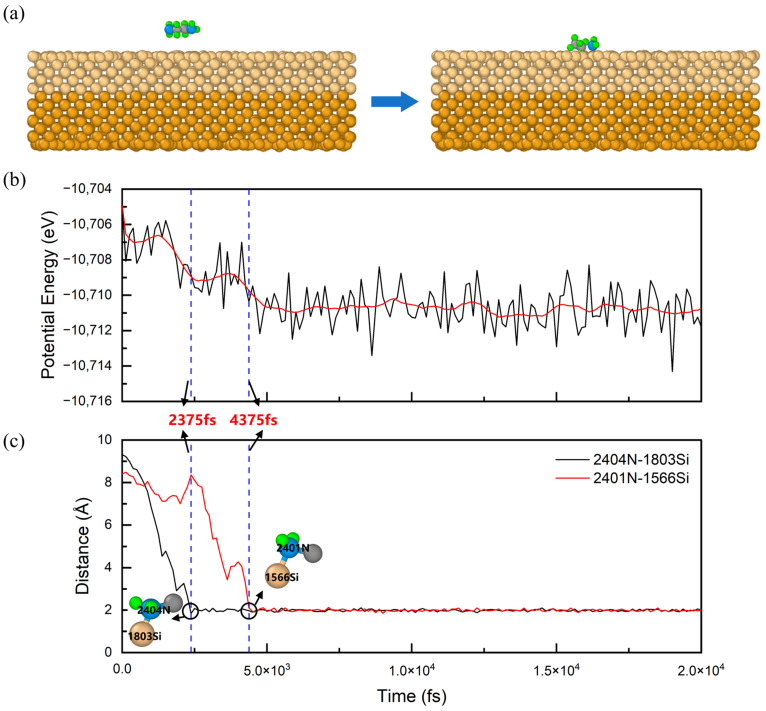
(**a**) The schematic diagram of the atomic structure of the EDA–Si system before (**left**) and after (**right**) the MD simulation. (**b**) The potential energy curve for the EDA–Si system. The red line is the smoothed potential energy curve. (**c**) Internal distance curves of the N–Si atom pairs during the adsorption. The two blue dotted lines in (**b**,**c**) correspond to the timepoints of the adsorption of the two N atoms in EDA, respectively. The prefix number on the atom represents the ID number of the atom in the simulation system. The corresponding atomic species of the various colored spheres are shown in Figure 3.

Figure 6a shows the schematic atomic structure of the EDA–Si system before and after adsorption. As can be seen in Figure 6a, the EDA molecule approached the silicon wafer and adsorbed onto the Si(1, 0, 0) surface. Figure 6b shows the potential energy curve of the EDA–Si system during the adsorption. There are two large decreases in the potential energy curve at 2375 and 4375 fs, corresponding to the adsorption processes of the two N atoms in EDA. The curve then displays a steady fluctuation, which is mainly caused by temperature fluctuation. To gain insight into the adsorption process of N atoms, we chose 2401N–1566Si and 2404N–1803Si (the number preceding the element symbol represents the ID of the atom in the simulation system) as atom pairs that would bond in the simulation, and we plotted the distance curve of the N–Si pairs during the adsorption, as displayed in Figure 6c. The distance curves also show that the adsorption times of 2404N and 2401N were around 2375 and 4375 fs, respectively. As the adsorption began, the atomic distance rapidly decreased with the proximity of the atomic pair, and after adsorption the N–Si atom pair distance stabilized. The final average distances of the 2404N–1803Si pair and the 2401N–1566Si pair were 1.997 and 1.986 Å, respectively. The potential energy curves of the remaining polyamine–Si systems during adsorption, along with the distance curves of atom pairs for the other polyamines, are detailed in Figures S1b–S4b.

**Figure 7:**
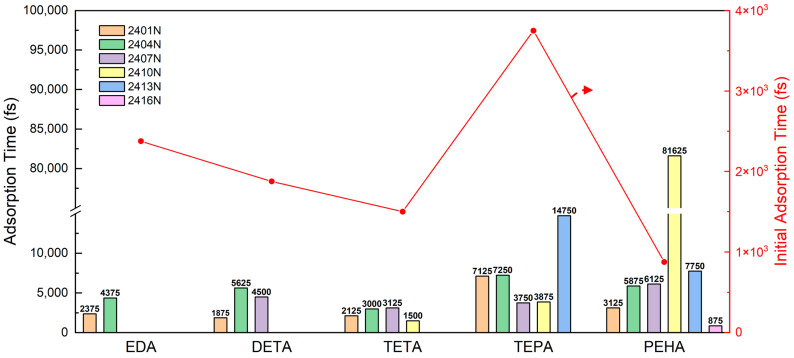
Adsorption times corresponding to N atoms on each type of polyamine. The number in front of the atom is the atomic ID in the simulation. The red folded line corresponds to the initial adsorption time of each polyamine, and the red arrow indicates that the values on the folded line are read from the right ordinate.

The average values of stable distances for the adsorbed atom pairs in the simulations of the five polyamines are listed in Figures S1c–S4c. Regardless of the type of polyamine, all of the Si–N bond lengths formed by adsorption were greater than those of the single Si–N bond of the conventional compound species [44,45], indicating that the adsorbed polyamines can be easily removed by external force. Figure 7 exhibits the adsorption times for all of the N atoms in the five types of polyamines, and the adsorption time of the first adsorbed N atom on the polyamine was taken as the initial adsorption time. The change in the initial adsorption time for different polyamines is shown as the red folded line in Figure 7. As the quantity of -CH_2_CH_2_NH- increases, the initial adsorption time of the polyamine is generally advanced. However, the time required for subsequent N atoms to adsorb becomes increasingly longer, because the molecule with one site adsorbed needs more time to adjust its configuration, making the adsorption of the remaining N atoms more difficult. The adsorption process of long-chain polyamines is roughly as follows: the head or tail of molecules preferentially adsorbs, and then the molecule forms a loop configuration and, finally, tapers to a train configuration. The adsorption behavior of TEPA differs, with a delayed initial adsorption time and preferential adsorption of N atoms in the middle of the molecule. This is presumed to be because the three-dimensional structural undulation of TEPA is greater than that of PEHA.

### 3.2. Multimolecular Adsorption of Polyamines

**Figure 8:**
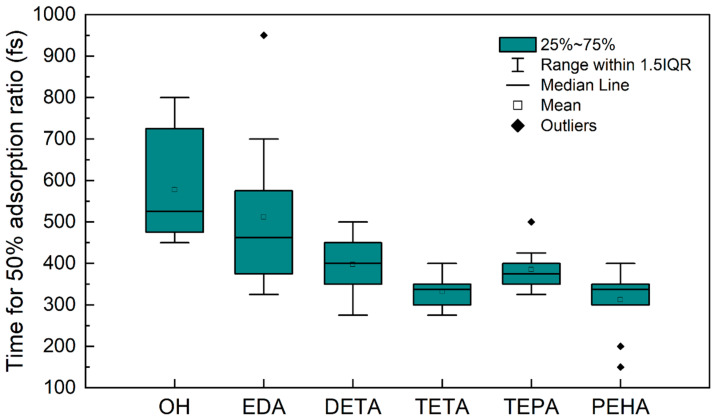
Boxplots of the time required for OH- and five polyamines (EDA, DETA, TETA, TEPA, and PEHA) to reach 50% adsorption on the Si surface.

Compared to unimolecular adsorption, the adsorption of multimolecular clusters may reflect the specific adsorption behavior of each type of polyamine, so we performed multimolecular adsorption simulations to observe the adsorption rate changes of different polyamines. Ten parallel simulations were run for each polyamine, and each simulation used a randomly constructed molecular layer containing ten polyamines. Figure 8 presents a boxplot of the time required for the five types of polyamines and OH^−^ to reach 50% adsorption on the Si(1, 0, 0) surface. As the quantity of -CH_2_CH_2_NH- in the polyamine increases, the adsorption rate of the molecular cluster initially accelerates and then converges, and the fluctuation in the adsorption rate also gradually decreases. The results of the unimolecular simulations of polyamines presented in Section 3.1 showed that an increase in the quantity of -CH_2_CH_2_NH- produces more adsorption sites, which causes the multimolecular simulations to be less affected by random initial positions and somewhat reduces the differences between the parallel simulations. The adsorption rate trend of the polyamines from the multimolecular simulations was consistent with the trend of initial adsorption time changes obtained from the unimolecular simulations. In addition, the adsorption rates of all five polyamines were significantly higher than that of OH^−^, implying that the polyamine additives have a preference to interact with the silicon surface during the Si CMP process.

**Figure 9:**
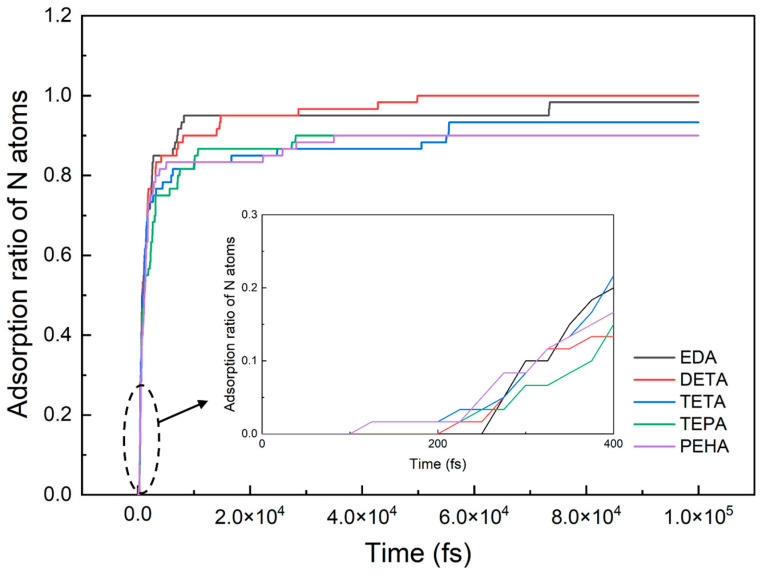
N atom adsorption ratio curves for the five types of polyamines. The inset shows the adsorption ratio curves from 0 to 400 fs.

We also performed adsorption simulations for the five polyamines while keeping the N content of the molecular layer consistent. From EDA to PEHA, the corresponding molecular numbers were 30, 20, 15, 12, and 10, respectively. The curves of the N atom adsorption percentage with simulation time for the different polyamines are displayed in Figure 9. As can be seen from the inset in Figure 9, in the range of 0–400 fs, the initial onset adsorption times for the polyamines were positively correlated with the quantity of -CH_2_CH_2_NH- in the polyamine, which is also consistent with the simulated results of the initial adsorption times in Section 3.1. Figure 9 shows that at the beginning of the simulations, the N atom adsorption ratios of all polyamines grew rapidly, exceeding 80% at 10 ps, and then the growth of the N atom adsorption ratios slowed down and differences in the adsorption ratios of the five polyamines appeared. The final N atom adsorption ratios of the five polyamines showed a decreasing trend with increasing chain length. According to the schematic atomic structure evolution (see Figure S9) in the simulations, the above trend was due to the distortion of the long-chain polyamines in the limited space during the adsorption process, making it difficult for some N atoms to adsorb onto the Si surface.

### 3.3. Effect of Polyamines on the MRR of Si CMP

**Figure 10:**
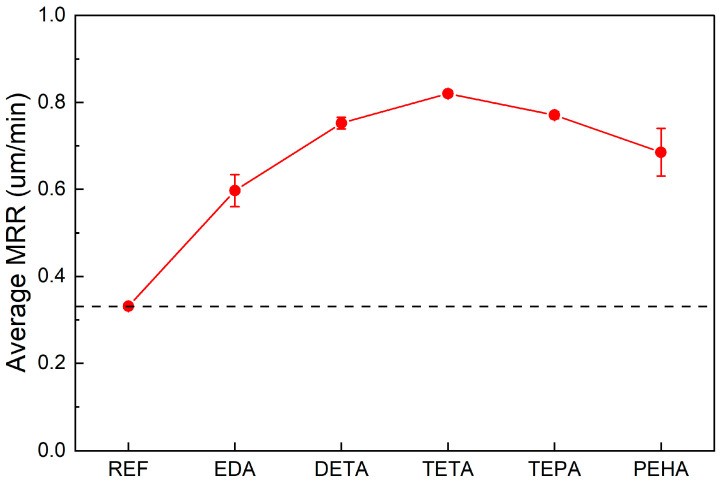
Effects of polyamines on the MRR of Si CMP.

**Figure 11:**
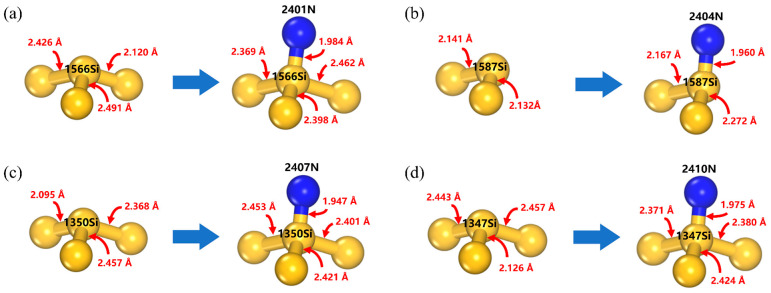
Changes in surface Si–Si bond lengths before and after TETA adsorption: (**a**) 2401N adsorption, (**b**) 2404N adsorption, (**c**) 2407N adsorption, and (**d**) 2410N adsorption. The yellow atoms correspond to Si, the blue atoms correspond to N, and the number preceding the atom indicates its atomic ID.

To further understand how polyamines affect the polishing rate of silicon CMP, CMP experiments were performed using the slurry samples numbered ‘Slurry 1–6’ in Section 2.3. The effects of the five polyamines on the MRR are shown in Figure 10. All of the MRR values were averaged from three CMP experiments. The reference slurry showed an MRR of 0.331 um/min. The addition of the five types of polyamines increased the polishing rate of the silicon wafers by 80–148% compared to the reference slurry, with MRR values of 0.597, 0.752, 0.820, 0.771, and 0.685 um/min, respectively. It can be seen that for EDA, DETA, and TETA, the effect of MRR enhancement showed a positive correlation with the adsorption rates obtained from the previous simulations, indicating that the adsorption rate of polyamine on the silicon surface is an important factor in improving the MRR of Si CMP. To further elucidate the influence mechanism of polyamines on the polishing rate of silicon CMP, the bonding information of the polyamine adsorption simulations was analyzed. The changes in the Si–Si bond lengths between the surface Si atoms before and after the adsorption of the TETA molecules are demonstrated in Figure 11 (see Figures S5–S8 for the effects of the adsorption of other polyamines on surface Si–Si bonds). It can be observed that, during the simulations, the adsorption of polyamines resulted in the stretching of the original surface Si–Si bonds (bond lengths of about 2.10~2.18 Å) by 7~18%, facilitating the removal of surface silicon atoms through the subsequent mechanical wear action of abrasives in the CMP process. Water on the silicon surface mainly produces OH^−^ to attack the Si–Si bonds [20], whereas simple adsorption of water molecules does not significantly affect the Si–Si bonds. In contrast, polyamines have a faster adsorption rate and the ability to stretch and weaken Si–Si bonds, making the Si atoms easier to remove; therefore, polyamines can increase the polishing rate of Si CMP.

**Figure 12:**
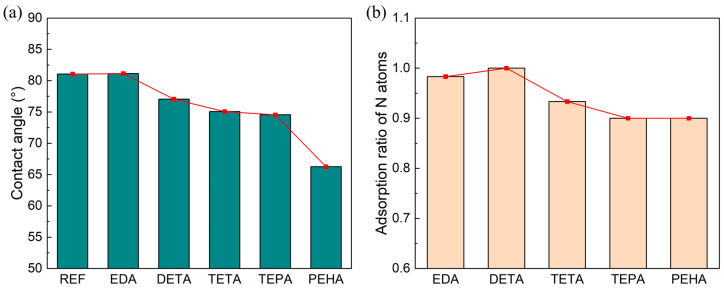
(**a**) Contact angle test results of polishing solutions containing different polyamines with silicon wafers treated with hydrofluoric acid. (**b**) The proportion of N atoms that can adsorb to the silicon surface with the same N contents of the adsorption layer in the multimolecular adsorption simulation.

Contact angle tests were performed to evaluate the adsorption effect of polyamines on the silicon surface. As shown in Figure 12a, the contact angle of the polishing solution on the wafer surface gradually decreases with the increase in the quantity of -CH_2_CH_2_NH- in the polyamine. This indicates that polyamines with more amine groups can make the silicon surface more hydrophilic. The data of the contact angle tests verify the MD results, showing that the adsorption rate and the adsorption strength of polyamines can be enhanced by increasing the number of amine groups.

The MRR of TEPA and PEHA shows a decreasing trend, suggesting that multiple factors affect the MRR in the CMP process. The results of the multimolecular simulations in Section 3.2 show that the number of N atoms that can be adsorbed on the silicon surface by the TEPA and PEHA surfaces is significantly reduced, as shown in Figure 12b. This leads to a weakening of the stretching effect of the Si–Si bonds on the surface, impeding subsequent corrosion and friction removal. Additionally, the long-chain polyamines form a dense adsorption layer on the silicon surface, severely hindering the mechanical friction between the abrasive and the silicon wafer, which also leads to a reduction in the MRR.

### 3.4. Effect of Polyamines on the Surface Quality of Si CMP

**Figure 13:**
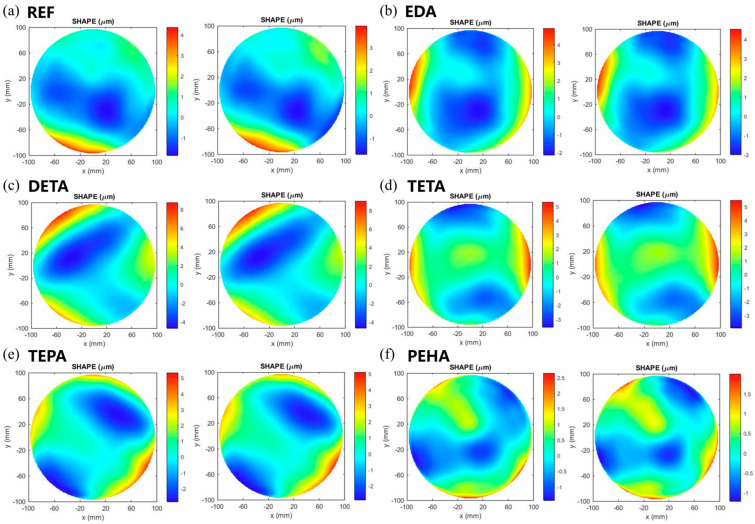
Surface morphology of silicon wafers before (**left**) and after (**right**) polishing with polishing slurries containing different polyamines: (**a**) reference (REF), (**b**) EDA, (**c**) DETA, (**d**) TETA, (**e**) TEPA, and (**f**) PEHA. Blue areas indicate depressions and red areas indicate protrusions.

**Figure 14:**
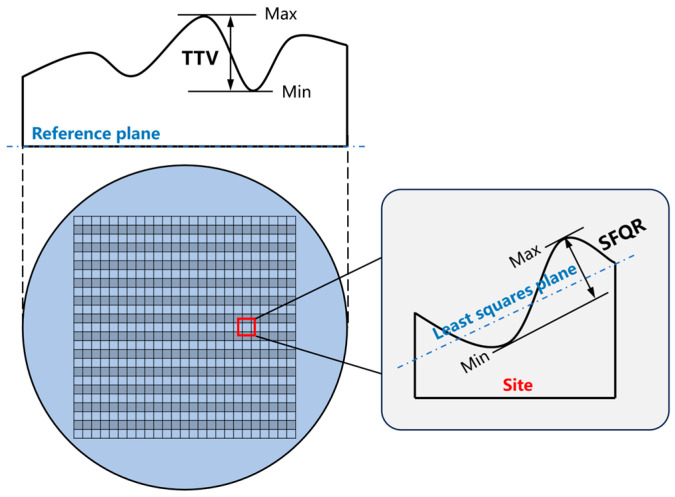
Flatness parameters of silicon wafers: TTV and SFQR.

Additionally, the surface quality after polishing with polyamines was also characterized in this work. Figure 13 illustrates the surface morphology of silicon wafers before and after polishing. The polyamines have almost no effect on the topographic distribution of the wafers. Therefore, the flatness parameters, mainly TTV (total thickness variation) and SFQR (site flatness, front-surface, least-squares fit (site), range), were used to further evaluate the surface quality. The definitions of these two parameters are shown in Figure 14.

**Figure 15:**
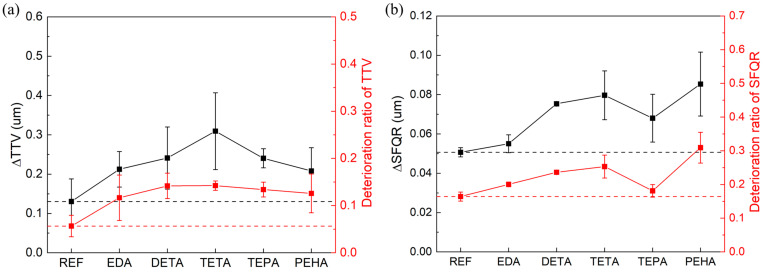
(**a**) ΔTTV and (**b**) ΔSFQR of silicon wafers after polishing. The red curve in the graph indicates the trend in the deterioration ratio of TTV and SFQR. The black and red dashed lines in the figure indicate the values of ΔTTV or ΔSFQR and their deterioration ratios, respectively, for the reference group.

The change values of TTV and SFQR before and after polishing, i.e., ΔTTV and ΔSFQR, were used to evaluate the changes in the wafer flatness, as shown in Figure 15. In terms of ΔTTV, the value obtained with the reference slurry was 0.130 um, while the values for the polyamine-containing slurries increased to 0.212 (EDA), 0.241 (DETA), 0.309 (TETA), 0.240 (TEPA), and 0.208 (PEHA) um. For ΔSFQR, compared to the reference value of 0.051 um, polyamines also resulted in an increase, reaching 0.055 (EDA), 0.075 (DETA), 0.080 (TETA), 0.068 (TEPA), and 0.085 (PEHA) um. It is comprehensible that increase in anisotropic etching through the addition of chemicals could result in a slight deterioration in the global and local flatness of the silicon wafer. Relative to the reference slurry, the increases in the percentage of TTV deterioration caused by the slurries with polyamines were all less than 10%, and the increases in the percentage of SFQR deterioration were all less than 15%, suggesting that polyamine has a weak deterioration effect on the surface quality of wafers.

## 4. Conclusions

In this study, the adsorption mechanism of polyamines on the Si(1, 0, 0) surface was investigated using the MD simulation method based on a reactive force field. Polyamines can adsorb on the Si surface and form weak Si–N bonds. From EDA to PEHA, the adsorption rate of these polyamines shows a tendency of increasing and then leveling off as the number of main-chain amine groups increases, i.e., as the number of adsorption sites increases. Furthermore, we explored the effect of polyamine adsorption on the surface Si structure, and we found that the adsorption of polyamines can stretch the surface Si–Si bonds to some extent, facilitating easier removal of Si atoms by mechanical friction. The faster adsorption rate of polyamines compared to water and hydroxide (two main components of the CMP slurry), combined with their ability to weaken Si–Si bonds, resulted in an improved MRR. CMP experiments confirmed the positive correlation between the adsorption rate of polyamines and the polishing rate of Si CMP, and further found that the enhancement of MRR is somewhat weakened due to an increase in the number of amine groups.

Compared to the existing research, this work has demonstrated the chemical impact of polyamines on the surface of silicon wafers at the atomic level, as well as their effect on the subsequent mechanical wear, and revealed the mechanism by which polyamines enhance the polishing rate. This provides valuable insights for the development of additives that can expedite the formulation of polishing solutions. The methods and concepts utilized in this study can also be extended to investigate the interactions between chemicals and substrate materials in the domains of CMP, cleaning, and etching. However, this study was limited by the existing force field, which cannot account for the complex polishing fluid environment and the mechanical friction process. These research topics will be explored in our future studies.

## Data Availability

Data are contained within the article.

## Supplementary Materials

The following supporting information can be downloaded at: https://www.mdpi.com/article/10.3390/nano14010127/s1, Figure S1: (a) The schematic diagram of the atomic structure of the DETA–Si system before (left) and after (right) the MD simulation. (b) The potential energy curve for the DETA–Si system. The red line is the smoothed potential energy curve. (c) Curves of the internal distances of the N–Si atom pairs. Figure S2: (a) The schematic diagram of the atomic structure of the TETA–Si system before (left) and after (right) the MD simulation. (b) The potential energy curve for the TETA–Si system. The red line is the smoothed potential energy curve. (c) Curves of the internal distances of the N–Si atom pairs. Figure S3: (a) The schematic diagram of the atomic structure of the TEPA–Si system before (left) and after (right) the MD simulation. (b) The potential energy curve for the TEPA–Si system. The red line is the smoothed potential energy curve. (c) Curves of the internal distances of the N–Si atom pairs. Figure S4: (a) The schematic diagram of the atomic structure of the PEHA–Si system before (left) and after (right) the MD simulation. (b) The potential energy curve for the PEHA–Si system. The red line is the smoothed potential energy curve. (c) Curves of the internal distances of the N–Si atom pairs. Figure S5: Changes in surface Si–Si bond lengths before and after EDA adsorption: (a) 2401N adsorption, (b) 2404N adsorption. The yellow atoms correspond to Si, and the blue atoms correspond to N. Figure S6: Changes in surface Si–Si bond lengths before and after DETA adsorption: (a) 2401N adsorption, (b) 2404N adsorption, and (c) 2407N adsorption. The yellow atoms correspond to Si, and the blue atoms correspond to N. Figure S7: Changes in surface Si–Si bond lengths before and after TEPA adsorption: (a) 2401N adsorption, (b) 2404N adsorption, (c) 2407N adsorption, (d) 2410N adsorption, and (e) 2413N adsorption. The yellow atoms correspond to Si, and the blue atoms correspond to N. Figure S8: Changes in surface Si–Si bond lengths before and after PEHA adsorption: (a) 2401N adsorption, (b) 2404N adsorption, (c) 2407N adsorption, (d) 2410N adsorption, (e) 2413N adsorption, and (f) 2416N adsorption. The yellow atoms correspond to Si, and the blue atoms correspond to N. Figure S9: Schematic distribution of polyamines before and after the adsorption simulations: (a) EDA, (b) DETA, (c) TETA, (d) TEPA, and (e) PEHA.
