# Supplementary material for: Mechanism Exploration of the Effect of Polyamines on the Polishing Rate of Silicon Chemical Mechanical Polishing: A Study Combining Simulations and Experiments
Source: Nanomaterials (Basel). 2024 Jan 4;14(1):127. doi: 10.3390/nano14010127 (PMC10780713; doi:10.3390/nano14010127)
Supplement: Supplementary file 1 [file nanomaterials-14-00127-s001.zip › nanomaterials-2779694-supplementary.pdf]

1. Unimolecular adsorption simulations for polyamines

1.1. Potential energy curve and interatomic distance for polyamines during adsorption in MD simulations

Figure S1 illustrates the results of unimoleculare adsorption simulation for Diethylenetriamine (DETA). From the potential energy curve and the atom pair distance curves, it can be seen that the adsorption order of N atoms is: 2401N → 2407N → 2404N, and the corresponding adsorption time points are 1875, 4500 and 5625 fs.

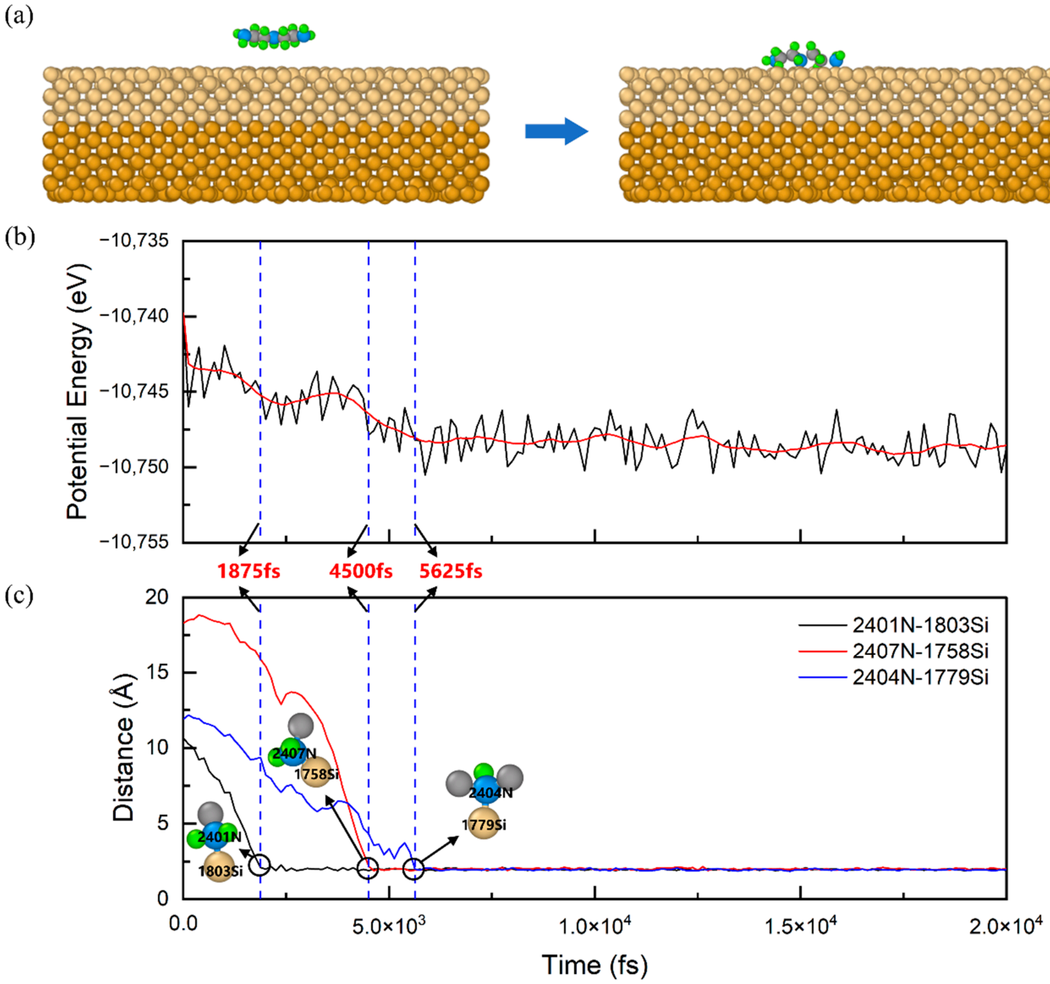

Figure S1. (a) The schematic diagram of the atomic structure of the DETA–Si system before (left) and after (right) the MD simulation. (b) The potential energy curve for DETA–Si system. The red line is the smoothed potential energy curve. (c) Curves of the internal distances of the N–Si atom pairs.

Figure S2 illustrates the results of unimoleculare adsorption simulation for Triethylenetetramine (TETA). From the potential energy curve and the atom pair distance curves, it can be seen that the adsorption order of N atoms is: 2410N → 2401N → 2404N → 2407N, and the corresponding adsorption time points are 1500, 2125, 3000 and 3125 fs.

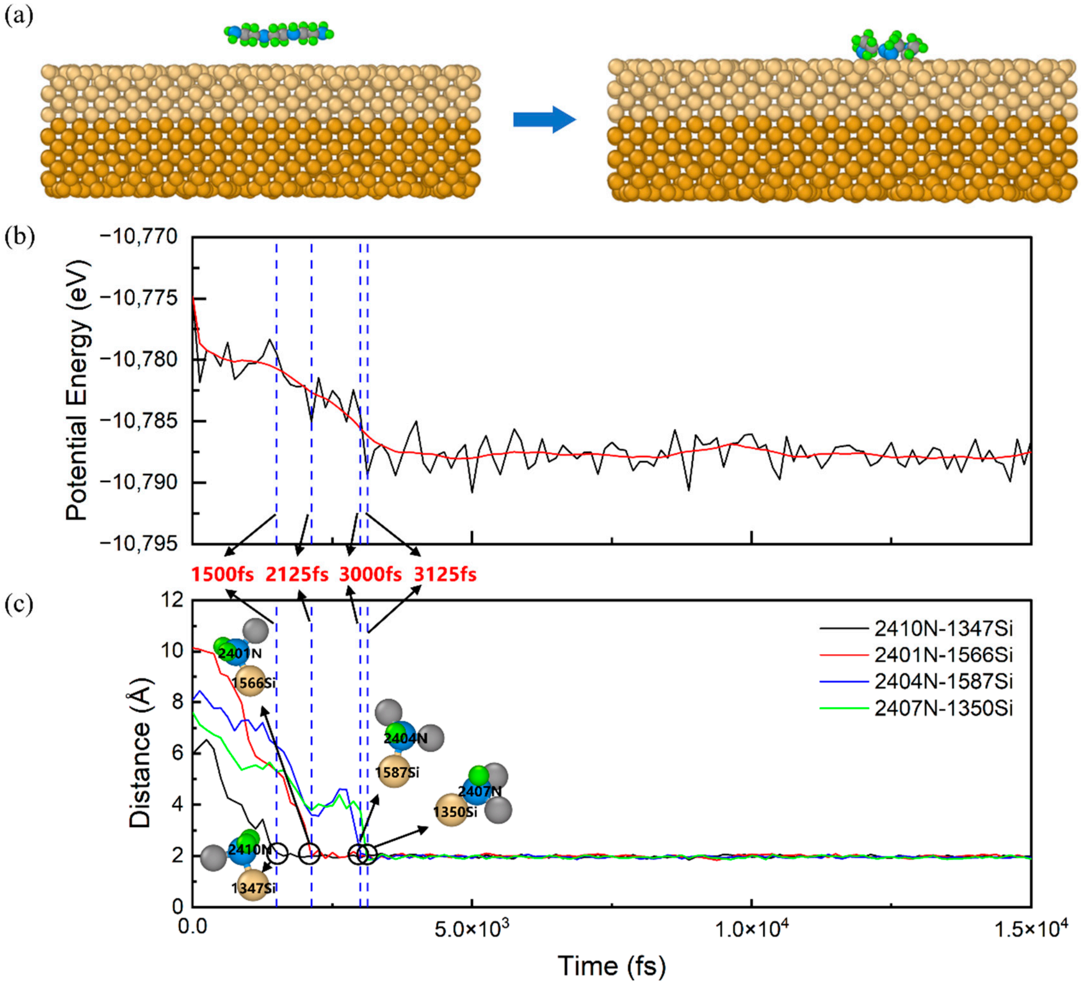

Figure S2. (a) The schematic diagram of the atomic structure of the TETA–Si system before (left) and after (right) the MD simulation. (b) The potential energy curve for the TETA–Si system. The red line is the smoothed potential energy curve. (c) Curves of the internal distances of the N–Si atom pairs.

Figure S3 illustrates the results of unimoleculare adsorption simulation for Tetraethylenepentamine (TEPA). From the potential energy curve and the atom pair distance curves, it can be seen that the adsorption order of N atoms is: 2407N → 2410N → 2401N → 2404N → 2413N, and the corresponding adsorption time points are 3750, 3875, 7125, 7250 and 14750 fs.

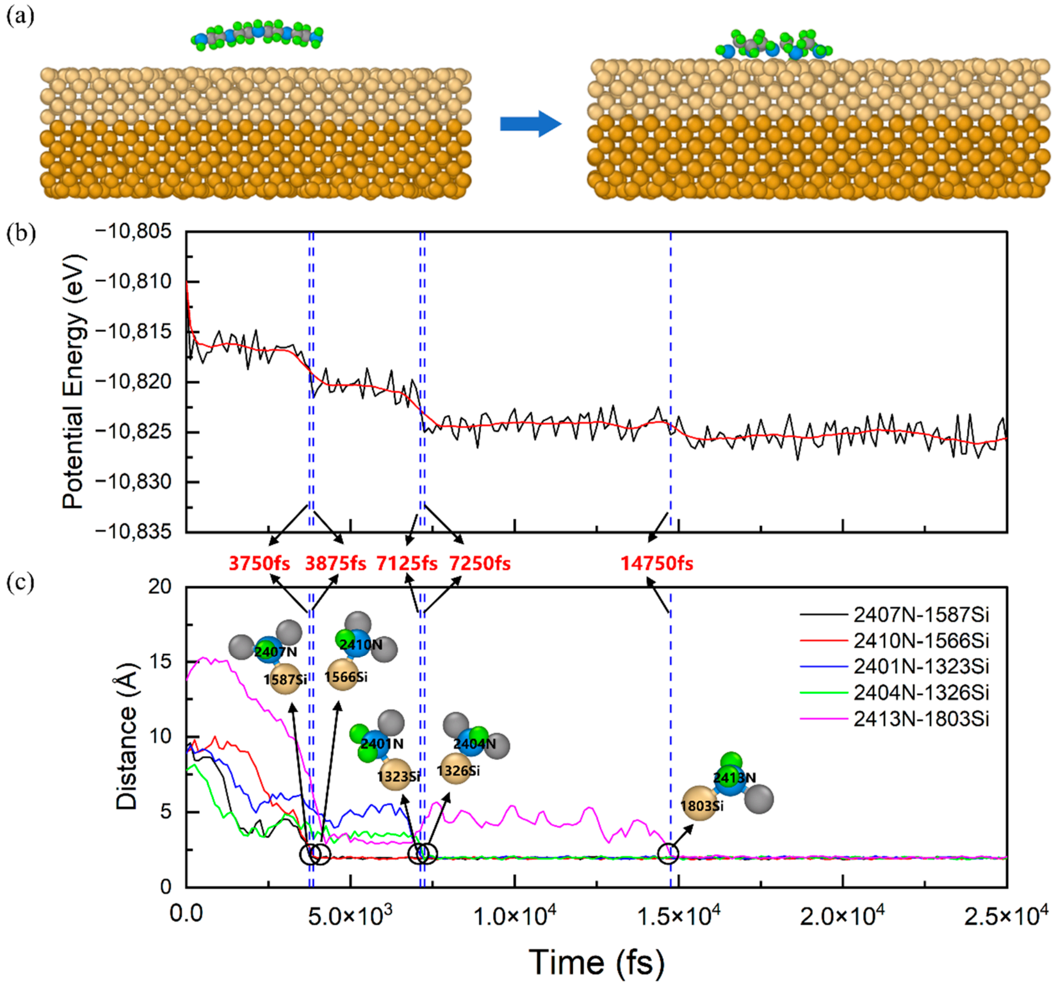

Figure S3. (a) The schematic diagram of the atomic structure of the TEPA–Si system before (left) and after (right) the MD simulation. (b) The potential energy curve for the TEPA–Si system. The red line is the smoothed potential energy curve. (c) Curves of the internal distances of the N–Si atom pairs.

Figure S4 illustrates the results of unimoleculare adsorption simulation for Pentaethylenehexamine (PEHA). From the potential energy curve and the atom pair distance curves, it can be seen that the adsorption order of N atoms is: 2416N → 2401N → 2404N → 2407N → 2413N → 2413N, and the corresponding adsorption time points are 875, 3125, 5875, 6125, 7750 and 81625 fs.

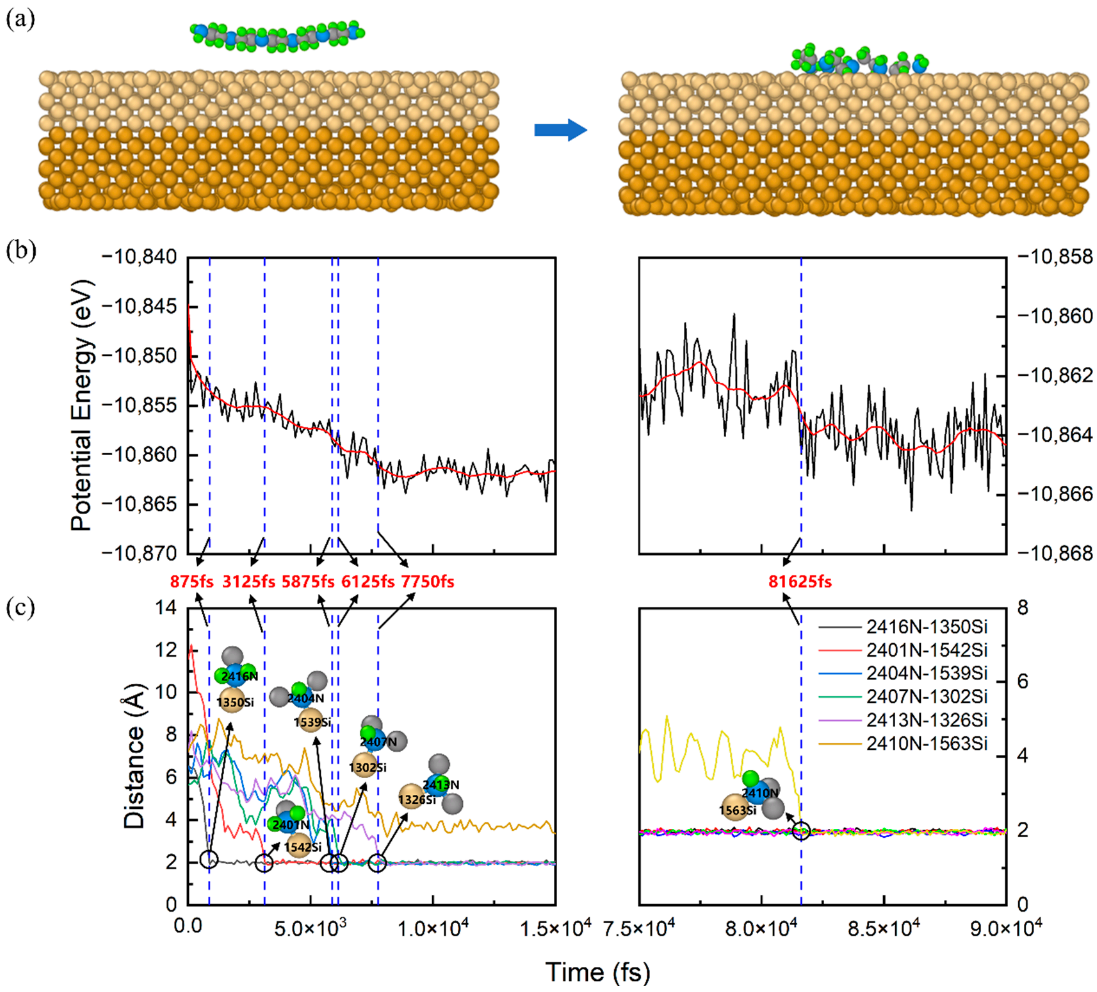

Figure S4. (a) The schematic diagram of the atomic structure of the PEHA–Si system before (left) and after (right) the MD simulation. (b) The potential energy curve for the PEHA–Si system. The red line is the smoothed potential energy curve. (c) Curves of the internal distances of the N–Si atom pairs.

1.2. Variations of surface Si-Si bonds lengths during adsorption simulation

From the bonding information, we obtained the changes in the surface Si-Si bond lengths before and after unimolecular simulations of EDA, DETA, TEPA and PEHA, as shown in Figures S5-S8. It can be seen that the adsorption of polyamines can stretch the Si-Si bonds, making the surface silicon atoms easy to remove.

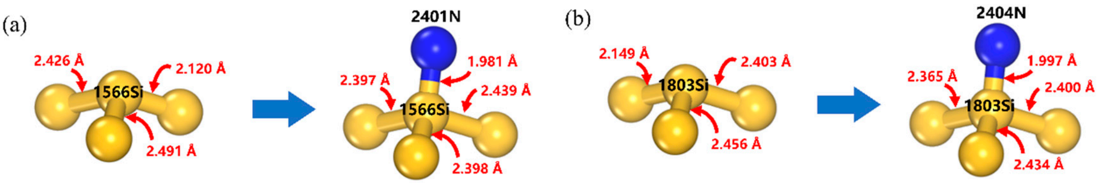

Figure S5. Changes in surface Si–Si bond lengths before and after EDA adsorption: (a) 2401N adsorption, (b) 2404N adsorption. The yellow atoms correspond to Si, and the blue atoms correspond to N.

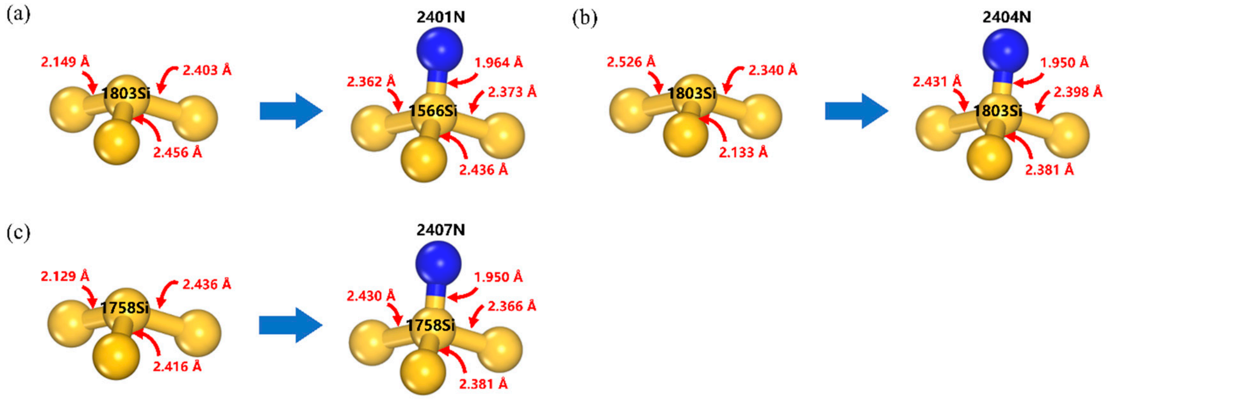

Figure S6. Changes in surface Si–Si bond lengths before and after DETA adsorption: (a) 2401N adsorption, (b) 2404N adsorption, and (c) 2407N adsorption. The yellow atoms correspond to Si, and the blue atoms correspond to N.

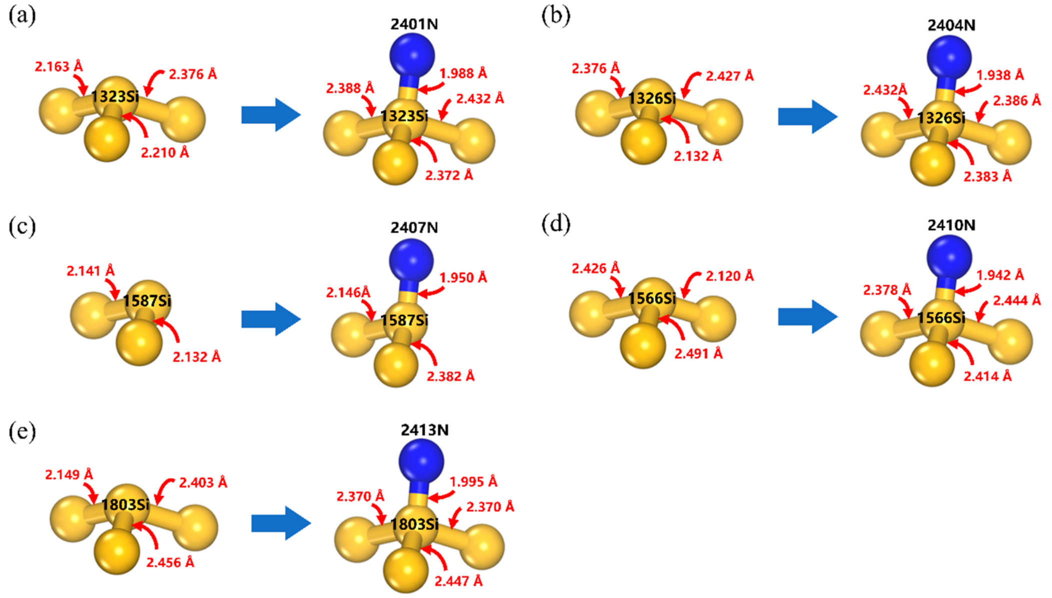

Figure S7. Changes in surface Si–Si bond lengths before and after TEPA adsorption: (a) 2401N adsorption, (b) 2404N adsorption, (c) 2407N adsorption, (d) 2410N adsorption, and (e) 2413N adsorption. The yellow atoms correspond to Si, and the blue atoms correspond to N.

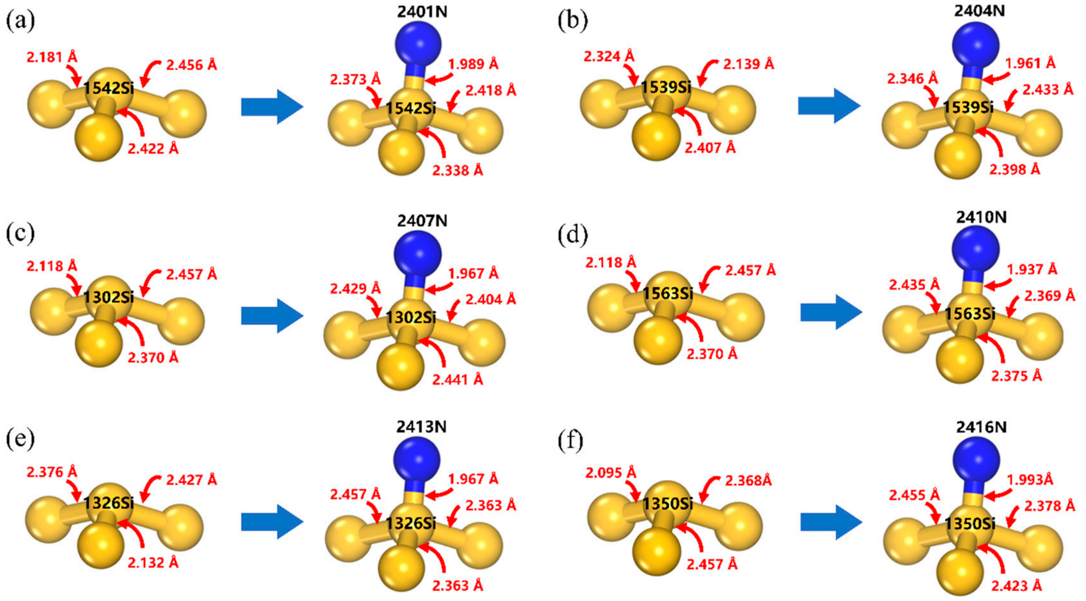

Figure S8. Changes in surface Si–Si bond lengths before and after PEHA adsorption: (a) 2401N adsorption, (b) 2404N adsorption, (c) 2407N adsorption, (d) 2410N adsorption, (e) 2413N adsorption, and (f) 2416N adsorption. The yellow atoms correspond to Si, and the blue atoms correspond to N.

Multimolecular adsorption simulations of polyamines with the same N content

Figure S9 shows the schematic distribution of polyamines with the same N content before and after the adsorption simulation. As the molecular chain grows, the molecule will twist when adsorbed on the Si surface, making it more difficult for the occurrence of adsorption at all sites on the molecule.

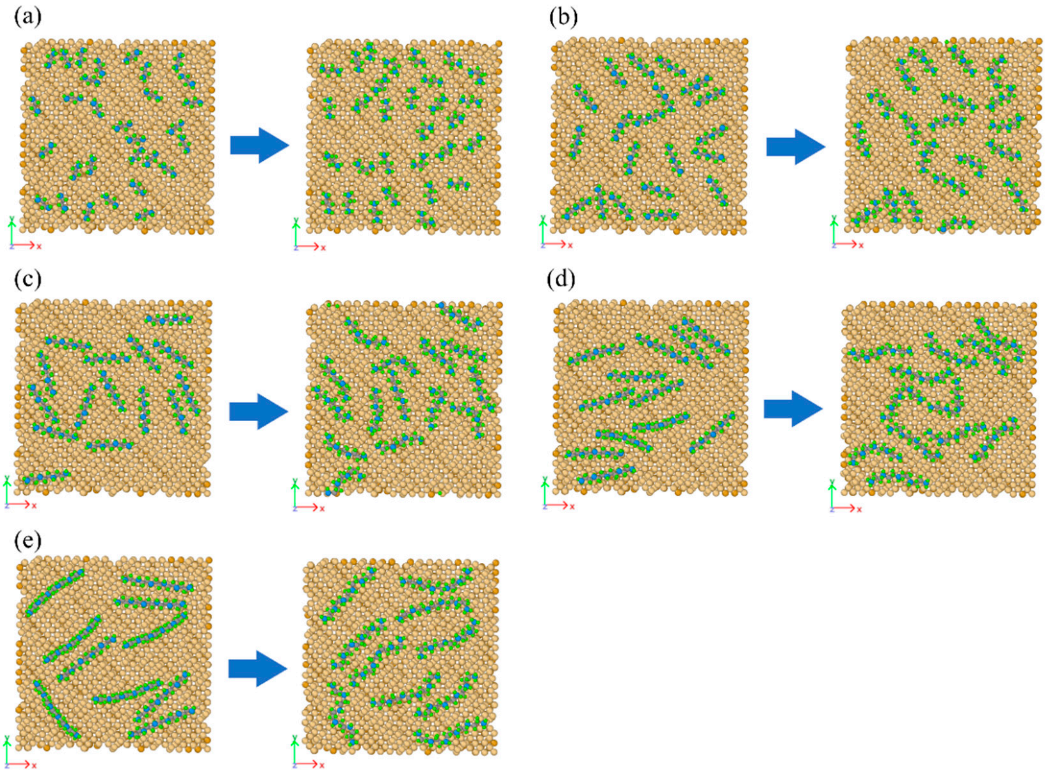

Figure S9. Schematic distribution of polyamines before and after the adsorption simulations: (a) EDA, (b) DETA, (c) TETA, (d) TEPA, and (e) PEHA.
